# Gut Microbiota and Energy Homeostasis in Fish

**DOI:** 10.3389/fendo.2019.00009

**Published:** 2019-01-24

**Authors:** Robyn Lisa Butt, Helene Volkoff

**Affiliations:** Departments of Biology and Biochemistry, Memorial University of Newfoundland, St. John's, NL, Canada

**Keywords:** fish, microbiota, feeding, energy, regulation

## Abstract

The microorganisms within the intestinal tract (termed gut microbiota) have been shown to interact with the gut-brain axis, a bidirectional communication system between the gut and the brain mediated by hormonal, immune, and neural signals. Through these interactions, the microbiota might affect behaviors, including feeding behavior, digestive/absorptive processes (e.g., by modulating intestinal motility and the intestinal barrier), metabolism, as well as the immune response, with repercussions on the energy homeostasis and health of the host. To date, research in this field has mostly focused on mammals. Studies on non-mammalian models such as fish may provide novel insights into the specific mechanisms involved in the microbiota-brain-gut axis. This review describes our current knowledge on the possible effects of microbiota on feeding, digestive processes, growth, and energy homeostasis in fish, with emphasis on the influence of brain and gut hormones, environmental factors, and inter-specific differences.

## Introduction

### Microbiota/Microbiome

The microbiota can be defined as the collection of microorganisms that occupy a particular environment whereas the term “microbiome” refers to the collection of genomes of the microorganisms within the microbiota (1). These microbial communities include commensal, symbiotic, and pathogenic microorganisms (2). Multicellular organisms, including plants and animals live in close association with microorganisms, and harbor such complex microbial communities in and on themselves, from the skin surface to the gastrointestinal tract (GIT) (2). The microbiota may be contracted and developed through exposure to environmental factors. Given the potentially large impact of the microbiota on the host health, an increasing number of studies have been carried out to characterize and determine the mechanisms of action of these microbes.

The diverse microbial community that colonizes the GIT (gut microbiota) plays a critical role in modulating the host's physiology (3–5). The gut microbiota has lived in symbiotic association with the vertebrate host for millions of years, the host providing a nutrient-rich environment for the microbiota, and the microbiota providing metabolic, protective, and structural functions for the host (4–6). The gut microbiota is often considered as an “extra organ,” as it plays a key role in the intestinal development and physiology, as well as overall development, growth and health (3). Recent studies suggest that the gut microbiota is involved in energy homeostasis by regulating feeding, digestive and metabolic processes, as well as the immune response (1, 6–8). In particular, the gut microbiota influences the brain-gut axis, the bidirectional communication between the GIT and the brain (9–11), by affecting both gut and brain (12) and thus helps to maintain host homeostasis.

The function of the gut microbiota and the subsequent physiological responses of the host depend on the composition of the microbes that are present in the intestinal track (11). There is a wide variation in the composition of fish gut microbiota between species and individuals, but several phyla have been shown to be dominant, including *Proteobacteria, Firmicutes, Bacteroidetes, Actinobacteria*, and *Fusobacteria* (13). To date, most of the studies on gut microbiota have focused on mammals, in particular rodents, and in comparison, little is known about the host-microbe interactions in fish (5). There are several limitations to using mammalian models, including husbandry constraints, and the use of isogenic strains. Owing to their short life cycles and high offspring numbers, and their diversity in genetics, physiology and immunological features, which can be easily manipulated, fish may represent valuable models to study microbiota in vertebrates (14, 15). In addition, studies on fish gut microbiota may help improve welfare of fish and aquaculture practices. However, notable differences exist between mammals and fish with regards to metabolism and energy expenditure (2) and variations in host-microbe interactions and in contributions to maintaining host homeostasis could be expected between fish and mammals.

This review describes our current knowledge on the role of the fish gut microbiota in the regulation of host physiology, with emphasis on feeding, digestion and metabolism, as well as its influence on stress responses, reproduction and development, and immune responses. Environmental and host-specific factors affecting the fish gut microbiota composition and actions are also discussed, as well as future implications of fish gut microbiota manipulation and potential research directions for this growing field.

## Physiological Roles of Gut Microbiota

### Feeding/Digestion/Metabolism

Studies in mammals show that microorganisms within the GIT are involved in the regulation of appetite/ingestion, digestion, and metabolism (16–19). For example, germ-free mice lacking a gut microbiota are leaner than normal control mice even when consuming more calories (17). Furthermore, these mice have lower levels of appetite-regulating hormones such as leptin and ghrelin (17), indicating that the gut microbiota is involved in the regulation of appetite and metabolism. Microbial secretions, including specific metabolites such as short chain fatty acids (SCFAs), indoles, propionate, butyrate, and acetate (20) affect digestive processes and metabolism. The microbiota also interacts with GIT neurotransmitters [e.g., serotonin (21), and the catecholamines dopamine, norepinephrine (22)] and thus influence their effects on gastrointestinal (GI) motility, function and hormone release, as well as feeding behavior (23, 24). Conversely, serotonin and catecholamines released from enteric neurons can influence the microbiota present in the gut and alter release of cytokines and bacterial molecules (25).

**Figure 1:**
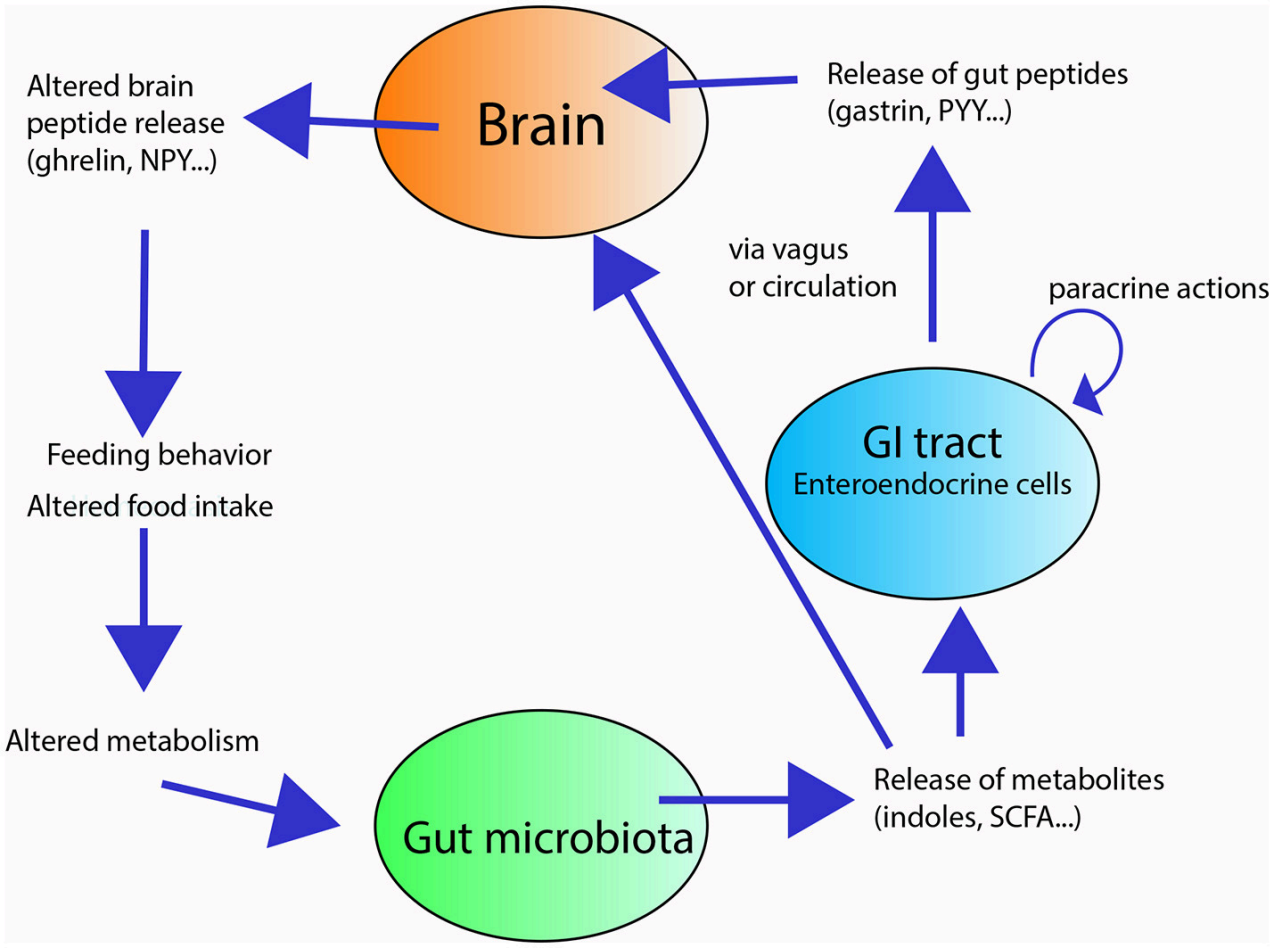
Overview of the gut-microbiota-brain axis in feeding and digestion. The gut microbiota (green circle) releases metabolites in response to substrates present in the gut lumen. These metabolites locally stimulate the enteroendocrine cells (blue circle) of the gastrointestinal tract (GIT) and/or reach the brain (orange circle). The stimulated enteroendocrine cells release gut peptides, which act locally in the GIT and affect brain feeding centers, altering neuropeptide release, and modifying feeding behavior and energy homeostasis.

Some of these metabolites can act on enterocytes and regulate their intestinal barrier function (26), absorptive capacity [e.g., monosaccharide absorption (27)], and nutrient uptake and storage [e.g., altered enzymatic activity in the gut and fat storage (28)], thus influencing metabolism [(e.g., cholesterol metabolism and adipogenesis) (29)]. Furthermore, metabolites from the gut microbiota can modify the secretory activity of enterocytes, thus affecting the production gut peptides that modulate gut motility and enzyme secretion (30, 31). For example, SCFAs receptors have been shown to interact with the enteroendocrine L cells containing the gut hormone Peptide YY (PYY) to influence the colonic PPY expression in rats (32), and further influence metabolism. These microbial compounds can also influence feeding behavior [e.g., (17, 33, 34)] directly, by entering the circulation and reaching the brain, or indirectly, by either by activating vagal terminals or by modulating the release of appetite-regulating gut peptides (e.g., CCK, ghrelin, gastrin), which in turn, affect the release of central appetite-regulating neuropeptides (e.g., neuropeptide Y, NPY; proopiomelanocortin, POMC) (33, 35, 36) (Figure 1). The exact mechanisms ruling the communication between the gut microbiota and the brain (termed the “microbiota-gut-brain axis”) and how changes within the gut microbiota may impact neuropeptide systems in the brain are still unclear (31).

To date, very few studies have been conducted in fish with regards to the influence of microbiota on feeding and metabolism, but they provide clues to some similarities with mammals in this regard.

The influence of microbiota on food intake has been examined in a few studies correlating feeding rates and changes in microbiota. However, results are inconsistent and difficult to compare, as several studies and several additives are used. For example, zebrafish fed with *Lactobacillus rhamnosus* have reduced appetite compared to control fish (37, 38). However, carp fed a diet supplemented with fructo-oligosaccharide (FOS) display changes in microbiota composition (increased levels of heterotrophic aerobic bacteria and lactic acid bacteria) but no changes in feeding rates compared to fish fed a control diet (39).

The potential effect of the gut microbiome on metabolism has been examined in a few fish species. In grass carp (*Ctenopharyngodon idella*), many biosynthesis, and metabolism pathways of carbohydrates, amino acids and lipids change as the composition of microbiota changes (40). In zebrafish, the colonization of the gut by microorganisms promotes epithelial absorption of fatty acids (41) and fish with intact microbiota have increased lipid accumulation in the intestinal epithelium, and increased expression of genes related to lipid metabolism compared to germ-free fish who lack microbiota (42). In addition, Japanese flounder (*Paralichthys olivaceus*) fed a diet supplemented with *Bacillus clausii* display higher weight gain, feed efficiency and growth performance compared to fish fed control diets (43). The authors suggest this could be attributed to increased food intake and improved nutrient digestibility (43). All this data suggests a strong influence of the microbiota in fish metabolism.

### Other Functions Related to Energy Homeostasis

#### Stress Response

The stress response is mediated by several hormones and is a result of the bi-directional communication between the brain and peripheral organs (2). Stress in fish can be caused by a number of environmental factors (including poor water quality, high levels of particulates, suboptimal photoperiod, oxygen levels, temperature), high population density, poor diet/ malnutrition, as well as transportation and handling (44).

When stress occurs, the hypothalamic-pituitary-adrenal (HPA) axis releases corticotrophin-releasing hormone (CRH), which stimulates the secretion of adrenocorticotropic hormone (ACTH) from the anterior pituitary, which stimulates the secretion of adrenal glucocorticoids to prepare the body to cope with stress (45).

In fish, as in mammals (45, 46), the microbiome affects the HPA axis, the stress response and behavior, in particular, anxiety-like and locomotor behaviors, which might in turn affect feeding behavior and energy homeostasis. For example, in zebrafish, enhancing the microbiota (by means of pro and prebiotics–see below) reduces anxiety-like behavior (47) and decreases the stress response, by lowering CRH expression and cortisol levels (48). Disruption of the gut microbiota might thus decrease the ability of the fish to forage for food and decrease feeding by increasing levels of stress hormones, which have been shown to inhibit feeding [e.g., rainbow trout *Oncorhynchus mykiss* (49); goldfish *Carassius auratus* (50)].

Conversely, stress can change the structure of the intestinal mucosa and induce alterations in the intestinal mucus, thus affecting absorption of nutrients as well as the gut immune system (or gut-associated lymphoid tissue, GALT), leading to infections by opportunistic pathogens (51). In fish, acute stress such as netting, induces an increased sloughing off of mucus and the removal of autochthonous bacteria which play a protective role against potential pathogens (52). Overall, stress results in modifications of gut microbiota and may alter immune response and increase the risk of colonization/invasion by pathogens and infection (2), which might decrease feeding rates, as seen in other fish [e.g., *goldfish* (53); chinook salmon (*Oncorhynchus tshawytscha*) (54, 55)]. However, different fish species may cope with stress in alternative ways, so that the effects of stress on gut microbiota may differ among species (52).

#### Reproduction/Development

##### Reproduction

Reproduction is closely related to energy homeostasis, as it is energetically costly, and can only be successfully accomplished when sufficient energy stores are available (56).

Studies have shown the gut microbiota may contribute to the development of gonads and subsequent reproductive success of the host. For example, when administered continuously from birth to sexual maturation, *Lactobacillus rhamnosus* alters the gut microbiota and accelerates larval development of zebrafish by improving growth and sex differentiation (57, 58). Adult female zebrafish treated with *L. rhamnosus* display an increase in the number of vitellogenic follicles and higher gonadosomatic indexes (GSI), higher numbers of ovulated eggs and higher expression levels of reproductive hormones (kisspeptinss, GnRH3, leptin) compared to control fish, therefore increasing the likelihood of reproductive success (58). Similarly, in ornamental livebearer fish species (59) and goldfish (60), supplementation of feed with probiotics increases GSI, fecundity and fry production of spawning females and length and weight of fry. Although the mechanisms mediating the actions of gut microbiota in host reproduction are still under investigation, it is likely that these mechanisms involve the regulation of feeding, food absorption and energy homeostasis.

##### Development

The development of tissues and organs which make up an animal is largely influenced by the presence and composition of the microbiota, in particular the development of the digestive and nervous systems (61), both crucial for appropriate ingestion and absorption of food.

The relationship between the fish gut microbiota and development of the host can be seen through clear patterns in the composition of gut microbiota during fish development (62). In grass carp (*Ctenopharyngodon idella*), bacterial communities vary between the eggs and the larvae, *Proteobacteria* and *Bacterioidetes* being dominant in the eggs and in the larvae, respectively, and bacterial diversity increases as the fish develops from egg to larvae (63). An increase in diversity has also been reported from the larval to the adult stage, as seen in grass carp, Chinese perch (*Siniperca chuatsi*) and southern catfish (*Silurus meridionalis*), also suggesting that the gut microbiota variation levels increase with fish development (62). In zebrafish, epithelial cell proliferation in the developing gut is stimulated by the presence of microbiota, providing direct evidence of the role of the gut microbiota in GIT development (64). Furthermore, the GIT of germfree zebrafish displays incomplete development and impaired function, which can be reversed by the inoculation of bacteria (14).

Evidence suggests that the microbiota is also involved in the neurological development, and is required for normal neurobehavioral development in the early life of the zebrafish. Fish with microbiota disruptions following antibiotic administration show abnormal locomotive activity (65), which might affect feeding behavior and foraging. The mechanisms ruling this interaction are still unknown.

#### Immune Responses

Pathogens might disrupt brain and intestinal functions and hamper feeding and growth (66). It has been proposed that the microbiota protects the host from colonization and proliferation of environmental pathogens, a process known as “colonization resistance” (67, 68). Although mechanisms behind this resistance are not clear, it has been suggested that commensal bacterial species compete with pathogens for niche space and produce and secrete antimicrobial peptides (67). Any disruption of the intestinal balance mucosa may thus lead to infections and activation of the GALT (69). The associated commensal microbiota of the mucosal immune system makes an important contribution to the immunity and metabolism of host fish, as the gut microbiota plays a major role in the development and maturation of the GALT (70, 71). For example, in both rainbow trout (70) and gilthead seabream (*Sparus aurata*) (72), administration of beneficial microorganisms (probiotics) enhances both the intestinal microbiota and the immune response.

## Factors Affecting Fish Gut Microbiota

**Figure 2:**
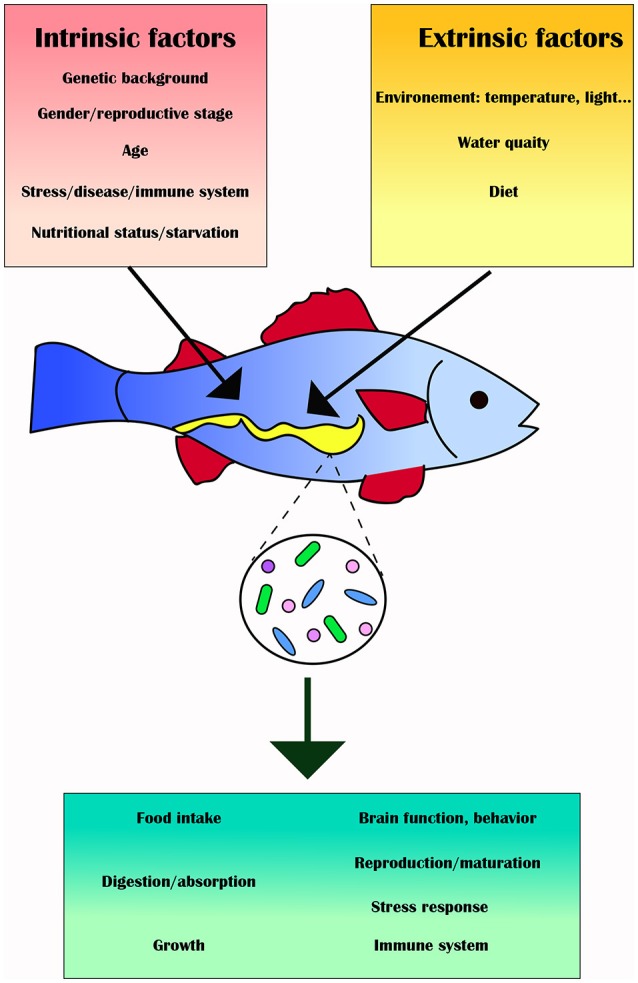
Intrinsic (red box) and extrinsic factors (yellow box) can alter the gut microbiota (green box) and its downstream effects on the fish host.

Biotic (e.g., genotype, physiological status, pathobiology, life style) and abiotic (e.g., environmental) factors may affect the fish gut microbiota and influence its composition and diversity, as well as its function and metabolic activity, thus affecting feeding, growth, energy storage and health of the fish (73) (Figure 2). This section will review these intrinsic and extrinsic factors and provide specific examples in which the gut microbiota of various fish has been altered as a result.

### Environmental Factors

Initially, fish embryos develop in a relative constant bacteria-free environment (within the egg or the mother). Fish are thus theoretically microbe-free at hatching, and gut microbes acquired post-hatch originate from surrounding environments (74). After hatching, fish are submitted to changing environmental factors (e.g., water composition and quality, and temperature), which can greatly influence the gut microbiota throughout their lifespan (52).

For example, the composition of gut bacteria differs between fish inhabiting freshwater and marine ecosystems (52). Generally *Aeromonas* and *Pseudomonas* predominate freshwater fish, whereas in marine fish, *Vibrio* is the most common genus (52). In black molly (*Poecilia sphenops*), an increase in salinity induces changes in dominant bacterial taxa in the microbiomes (75) and in rainbow trout, an increase in temperature results in an increase in microbial growth (76).

The gut microbiota of a species can also fluctuate over short time scales such as within 1 day (77) and days, or longer time periods (months or years) and this may be the result of seasonal variations (78).

Seasonal changes, accompanied by changes in temperature might induce alterations in food consumption due to variations in nutrient loads in the water column (79). As a consequence, the composition of the microbiota may be associated with particular seasons. For example, the gut bacterial load of tilapia decreases in winter compared to other seasons (79), and in the gut of hybrid tilapia (*Oreochromis niloticus* × *Oreochromis aureus*), *Pseudomonas, Micrococcus*, and *Flavobacterium* are only present in the winter (79).

Rearing conditions have also been shown to influence the composition of the gut microbiota. For example, in Atlantic salmon, fish held in two different holding conditions (indoor recirculating aquarium facility and cage culture in an open freshwater “loch” environment) have different microorganism compositions within their gut microbiota (80).

Pollutants and toxins present in the environment may also influence the fish microbiota. For example, common carp (*Cyprinus carpio*), exposed to waterborne copper (81) and zebrafish exposed to polystyrene microparticles (82) display disturbances of the intestinal microbiota related to immunity, which increase their susceptibility to pathogens and inflammation (microbiota dysbiosis). Other kinds of environmental chemicals such as pesticides [e.g., (83)], heavy metals [e.g., (84)] and antibiotics [e.g., (85)], can induce gut microbiota dysbiosis associated with changes in the intestinal mucus layer and inflammation in fish, thereby reducing the ability of absorb nutrients.

### Host-Specific Intrinsic Factors

The variations in microbiota composition among fish might be due to several factors, including phylogeny, genetics and sex, age/life stages, and diet/feeding habits (86).

#### Genetics/Sex

Genetic background influences the gut microbiota and intra- and inter-specific variations in microbiota have been demonstrated. Interspecies differences in the composition of gut microbiota among individuals of the same species may be present. For example, in rainbow trout, some bacterial groups are associated with specific families, perhaps due to different habitats or different diets (87). Inter-specific differences bacterial community structure are also seen, even if species exposed to the same environment [e.g., four freshwater larvae of silver carp, grass carp, bighead carp, and blunt snout bream (88)].

The sex of the fish may influence the gut microbiota through sex-specific host-microbe interactions, diet preferences or immune responses (89). For example, differences in the gut microbiota between sexes of the threespine stickleback (*Gasterosteus aculeatus*) and Eurasian perch (*Perca fluviatilis*) have been reported (89), but the gut microbiota of the zebrafish is similar between sexes (61, 90). However, given the small number of published reports and the wide variation in studies, the underlying mechanisms are not yet understood.

There are still questions on whether genetics or the environment has greater influence on the gut microbiota. To date, host genetics has been considered the most influential in shaping the fish gut microbiota. Channel catfish (*Ictalurus punctatus*) and blue catfish (*I. furcatus*) raised under constant environmental and husbandry conditions have similar gut microbiota compositions, suggesting that a shared environment can overcome differences in host genetics (91).

#### Age/Sexual Maturity

Differences in the microbiota composition have been identified between juvenile and sexually mature individuals (44, 52). Zebrafish juveniles have higher bacterial richness in their gut microbiotas than elderly adult fish (44, 62), suggesting an increase response of gut microbiota to higher levels of circulating sex hormone levels in adult compared to juvenile fish (44). Furthermore, the GALT may interact differently with the gut microbiota in juvenile and mature zebrafish as this system is not fully developed in the juveniles (44). Similarly, in southern catfish, gut microbial diversity increases as the host ages (92).

Changes in the gut microbiota have also been shown during the early life development stages of fish. In Atlantic salmon (*Salmo salar*), intestinal microbiota compositions vary between embryonic stages, with embryonic communities having lower richness and diversity compared to those of hatchlings (93). In Malaysian Mahseer (*Tor tambroides*), larval, juvenile, and adult stages have higher gut microbiota diversity than fingerling and yearling stages (94).

It is possible that modifications in diet contribute to the differences between juvenile and mature fish gut microbiota. Fish of different ages and sexual stages might have different nutrient requirements and might adjust their diets and feeding rates to obtain adequate energy intake [e.g., in Gibel carp, juvenile fish require more proteins than pre-adults (95)]. As the diet and gut microbiota composition change with age, is likely that the nature of the contribution of the gut microbiota to host homeostasis also changes. However, further research is needed to explain this potential relationship between fish age, fish gut microbiota and host energy homeostasis.

#### Feeding Habits/Diet

Feeding habits can greatly influence the structure and composition of the gut microbiota (52). Gut bacterial diversity is generally lower in carnivores, and progressively increases in omnivores and herbivores (71). For example, the most abundant bacteria in herbivorous fish include *Clostridium, Citrobacter*, and *Leptotrichia, Cetobacterium* and *Halomonas*, in omnivorous fish, and *Clostridium, Cetobacterium*, and *Halomonas* in carnivorous species (96–98). This trend has been found in both marine and freshwater fish, suggesting that the trophic level is likely one of the most influential factor affecting the gut microbiota composition (86).

The gut microbiota can also vary within species of the same trophic level. For example, the gut microbiota of four herbivorous Asian carp species (silver carp, *Hypophthalmichthys molitrix*; bighead carp, *Hypophthalmichthys nobilis*; grass carp, *Ctenopharyngodon idella*; and common carp, *Cyprinus carpio*) reared in the same environmental conditions exhibits inter-specific differences, in particular with regards to the relative abundance of the cellulose degrading phyla *Firmicutes*, most probably due to species-specific diets (99).

A limited number of studies show that modifying the diet of the fish can result in alterations of the gut microbiota, but this is not always the case. Diets containing guar gum, (non-starch polysaccharide) fed to the omnivorous mullet, *Mugil liza* (100) or soy proteins to the carnivorous rainbow trout (101) induce alterations in the bacterial quantity and composition in the GIT. In zebrafish, administration of dietary nucleotides results in modifications of the microbiota and reduction in fatty acid oxidation in muscle and liver as well as lower inflammatory tone (102). However, in channel catfish, different diets with different protein sources including animal and plant meals only have minimal effects on gut microbiota (103).

Extreme dietary changes, such as fasting also shape the fish gut microbiota. During times of fasting, morphological changes in the GIT occur due to the reduced nutrient uptake, which may account for changes observed in the gut microbiota (101). Furthermore, a depletion of nutrients induces changes in gut microbiota composition to favor bacterial species/communities that use more diverse energy sources and are capable of survival under limited nutrient conditions (52). Microbiota gut diversity and richness are usually higher under feeding conditions than under fasting conditions as seen in zebrafish (58) and in leopard coral grouper (*Plectropomus leopardus*) (104). In grouper, the dominant phyla are *Proteobacteria* in fasting and *Firmicutes* in fed conditions (104) and in the Asian seabass, *Lates calcarifer*, fasting induces a significant enrichment of *Bacteroidetes* and a depletion of Betaproteobacteria (105).

The act of feeding itself can also influence the microbiota. In rainbow trout, a short time after feeding (3 h) is not sufficient to cause any significant changes in the bacterial composition, but can cause changes in species richness and relative abundance (106). Similarly, in Southern catfish, although the diversity remains the same, the relative abundance of bacterial phyla differs at 3, 12, and 24 h after feeding, suggesting that short-term food digestion may alter community structure, but has less effect on the microbial composition (92).

## Gut Microbiota Manipulation and Applications

The role of the fish gut microbiota in host physiology has become increasingly evident with growing research in this field. Several experimental methods have been used to assess the role of the fish gut microbiota via the manipulation of these communities, including gnotobiotic, antibiotic, probiotic and prebiotic, and symbiotic studies (Table 1).

**Table 1:** Fish gut microbiota via the manipulation techniques used in research (see references in text).

**Microbiota manipulation**	**Objective**	**Potential use**	**Advantages**	**Disadvantages**	**Examples of host species examined**	**Representative references**
Gnotobiotic	Establish a germfree (gut microbiota absent) host or a known microbiota composition (predefined microbiota)	Examine the effects of an absence of microbiota (germ free) on the host physiology or the effects of specific gut microorganisms and/or a predefine microbiota on the host	Control over multiple variables and analysis of host responses to specific changes in microbiota	Complex procedures required to produce and maintain gnotobiotes	Zebrafish	(52) (14)
Antibiotic	Inhibit or eliminate targeted gut microbiota bacteria and or bacterial pathogens	Bacterial disease prevention/treatment	Bacterial disease prevention/treatment	Potential to disrupt microbial communities and increase disease susceptibility, and bioaccumulation	Zebrafish, Mosquito fish Black molly	(107) (108) (109)
Probiotic	Establish beneficial gut microbiota bacteria	Use in aquaculture, improve fish health (digestion/growth), health management, disease prevention	Enhancement of immune function of the host, resistance to pathogens, and overall health	No known disadvantages. Probiotics are considered safe overall.	Zebrafish Rainbow trout Malaysia masheer	(110) (111) (112)
Prebiotic	Stimulate growth of probiotic bacteria	Use in aquaculture, improve fish health (digestion/growth), health management disease prevention	Enhancement of immune function of the host, resistance to pathogens, and overall health	No known disadvantages. Prebiotics are considered safe overall.	Rainbow trout Nile tilapia Common carp fry	(113) (114) (115) (116)
Symbiotic	Establish prebiotic and probiotic bacteria	Use in aquaculture, improve fish health (digestion/growth/immune system), health management, disease prevention	Improvement of immune response (better than probiotics alone) and increase in growth and feed utilization in host.	No known disadvantages. Symbiotics are considered safe overall.	Nile tilapia	(117)

### Gnotobiotic Fish

Gnotobiotic animals (or gnotobiotes) are animals with a known microbiota composition. These include germ-free (or axenic) animals and axenic animals that have been inoculated with known microorganisms. Studies involving gnotobiotic fish allow the control over many variables that affect the development of the microbiota and analysis of host responses to specific gut microorganisms (14, 52). The disadvantages of this type of study is the complex procedures involved in the production and maintenance of gnotobiotes (52).

### Antibiotics

Antibiotics (or anti-bacterials) can be considered environmental factors affecting the gut microbiota. In the aquatic environment, they may be found naturally or as pollutants discharged as metabolites [such as sulfamethoxazole (SMX) and oxytetracycline (OTC)] through feces or urine of treated humans or animals (107). Mosquitofish (*Gambusia affinis)* exposed to antibiotics display lower community diversity and taxonomic composition from both skin and gut microbiomes, compared to untreated fish (108) and in zebrafish, exposure to OTC results in a disruption of the intestinal microbiota (107). Antibiotics can be used to manipulate the microbiota, as they kill or inhibit the growth of specific bacteria. The administration of antibiotics does not completely eliminate gut microbiota communities but can cause significant changes in the microbial composition.

The use of antibiotics in aquaculture for disease prevention and treatment is common. However, antibiotics may disrupt the microbial communities and increase disease susceptibility (56, 118). Furthermore, antibiotics can bioaccumulate in animal tissues (56) and lead the development of drug-resistant bacteria, which can be passed along the food chain (119, 120). Due to the growing awareness of the disadvantages of antibiotics, strict regulations have been established in the aquaculture industry and alternative methods are being developed and tested (121). Treatment of fish with beneficial microorganisms (probiotics) is a promising solution to antibiotics, as these probiotics inhibit the colonization of potential pathogens by producing antibacterial peptides and competing for nutrients with detrimental bacteria (122). Probiotics may thus reverse the negative effects of antibiotics and improve fish health. For example, in black molly *Poecilia sphenops*, successful colonization of two probiotic species (*Phaeobacter inhibens* and *Bacillus pumilus*) reverses the negative impacts of antibiotics, and decreases mortality rates (109).

### Pro-, Pre- and Symbiotics

Probiotics are live or dead component of a microbial cells which confer a health benefit to the host through promoting beneficial intestinal bacterial species, whereas prebiotics are non-digestible food ingredients that selectively stimulate the growth of probiotics (73). The supplementation of probiotics and/or prebiotics into the diet of fish is believed to result in beneficial alterations of the gut microbiota and subsequent changes in metabolism and energy expenditure that are beneficial for the host (119, 123, 124). The administration of these supplements also enhance immune function of the host and increase its resistance to pathogens, enhancing general health and indirectly favoring feeding and growth (125). These effects highlight the potential for probiotics/prebiotics to enhance fish health by manipulating the fish gut microbiota.

Commonly used probiotics in aquaculture include members of the *Lactobacillus, Lactococcus, Leuconostoc, Enterococcus, Carnobacterium, Shewanella, Bacillus, Aeromonas, Vibrio, Enterobacter, Pseudomonas, Clostridium*, and *Saccharomyces* genera (56). Multispecies probiotics may be more effective than single-strain probiotics as different strains present in multispecies probiotics increase the chance of survival in the gut [as seen in rainbow trout (111)]. Probiotics increase the number of beneficial gut bacteria. For example, feeding fish with probiotics results in a higher abundance of core gut bacteria in zebrafish (110), and an increase in bacterial diversity in rainbow trout (111) and Malaysian mahseer (112).

Although exceptions exist, probiotics generally promote feed efficiency and growth in fish [e.g., tambaqui *Colossoma macropomus* (126), Japanese flounder (43), tilapia (123), carp (127), red seabream *Pagrus major* (128), trout (111)], likely by increasing nutrient absorption and perhaps feeding. An increase in absorption results from changes in intestinal morphology, with higher absorptive surface areas and higher microvilli densities in the intestine [e.g., tilapia (129), zebrafish (38), Malaysian mahseer (130)]. The effects on growth might be mediated by changes in the expression of growth-related genes. For example, following probiotics treatment, growth hormone (GH) expression levels increase in pituitary of Malaysian mahseer (112) and in liver of yellow perch (*Perca flavescens*) (131), and insulin-growth factor (IGF-1) expression (112) is upregulated in the liver of Malaysian mahseer (112) and yellow perch (131) and in body of European seabass (132).

Overall, the effects of probiotics on feeding have been little examined to date and remain unclear. In tambaqui (133), fish fed probiotics (*Bacillus subtilis*) and control diets have similar feeding ratios, and probiotics induce a reduction in appetite in zebrafish [*Lactobacillus rhamnosus* (38)] and an increase in food consumption in pacu [*B. subtilis* (134)]. The discrepancies in results are likely due to variations in the nature and doses of probiotics used. These alterations in feeding might be partially due to the modulation of the expression of genes related to appetite. In larval zebrafish, administration of the probiotic *Lactobacillus rhamnosus* induces a decrease in brain NPY expression and an increase in adipose tissue leptin expression (38), and in goldfish, ghrelin intestinal expression is down-regulated in *Lactobacillus aidophilus* fed fish compared those of fed control diets (135).

In comparison to probiotics, prebiotics have received less attention with regards to their potential for aquaculture. However, overall, studies show that prebiotics have beneficial effects on growth performance, digestive enzyme activity, as well as disease and stress resistance of the host (136). Common prebiotics used in fish include inulin, fructooligosaccharides (FOS), short-chain fructooligosaccharides (scFOS), and mannanoligosaccharides (MOS) (137). Prebiotics have been shown to increase growth performance and feed utilization in some fish [e.g., rainbow trout, (113, 114), pacu *Piaractus mesopotamicus* (138), Nile tilapia (115), Caspian roach (*Rutilus rutilus*) (139)], but this is not always the case. For example, growth performance is not affected in common carp fry (*Cyprinus carpio*) fed inulin (116) or in pacu fed β-glucan (134). The increase in growth seen in some studies might be in part due to modifications in the intestinal structure (i.e., increases in intestinal villi height and digestive enzyme activities), as seen following probiotics administration. An improved immune system function [e.g., rainbow trout (114), Caspian roach (139)] and improved stress response [zebrafish (48)] most probably also largely contribute to a better growth performance of the fish.

Prebiotics and probiotics may also be administered in combinations, known as synbiotics (140, 141). Studies show that synbiotics improve the survival and implantation and metabolism of probiotic health-promoting bacteria in the GIT (142). Synbiotics have been shown to increase growth performance and feed utilization in the host, which may be a result of providing the host with energy and nutrients, and/or enhanced digestion processes (143). For example, following the supplementation of a probiotic (*Bacillus licheniformis*) and a prebiotic (yeast extract), growth performance is increased in Nile tilapia (*Oreochromis niloticus*), and this is accompanied by an increase in feed intake and feed utilization (118). The combination of probiotics and prebiotics in the diet also results in better immune responses than probiotics alone [e.g., rainbow trout (117)].

## Conclusions

Several studies strongly suggest that the fish gut microbiota influences the overall health of the host fish with regards to overall physiology, digestion, stress response, reproduction, and the immune system.

Relatively few studies on the effects of the microbiota on energy homeostasis have been conducted to date and large variations exist between results, making them difficult to compare.

First, given the possible influence of genetics and the environment, and the low number of species examined, many more species need to be examined before conclusions can be made. Second, a variety of methods has been used for studying the fish gut microbiota, and the results obtained may vary depending on the experimental methods used, highlighting the need to develop appropriate standardized methods to describe fish microbiota (52). Studying the influence the gut microbiota may have on fish energy balance is challenging, as several different mechanisms of action are responsible, involving both local and endocrine pathways, different physiological systems (e.g., stress, immunity…) and molecules (hormones, metabolites…), and that all these systems interact (e.g., gut-microbiota-brain axis communication). Furthermore, each microorganism within the microbiota might have different actions. In addition, compared to terrestrial animals, fish are more exposed to constant environmental changes that could affect the microbiota.

Manipulating the gut microbiota of fish has great potential for aquaculture use to improve growth. However promising, the future of probiotics/prebiotics faces several challenges, including appropriate modes of treatment (oral, or in the water) and doses, the characterization of mechanisms of action of individual probiotic organisms, and quality control and regulation (144, 145). The fish model can also be useful to understand the gut microbiota in other vertebrate species such as humans. Zebrafish (58) and threespine stickleback (14) have been widely used as they are small fish that can be easily maintained in laboratory conditions, and have rapid development and generation times. In addition, their genomes are readily available and display structural and functional genetic similarities to humans.

Therefore, although progress has been done, much remains to be resolved using fish models for gut microbiota. Nonetheless, the research conducted to date has offered great insights into the mechanisms by which these communities are able to regulate the fish host, and provided insights into improving aquaculture practices, and better understanding the host microbe relationships among other vertebrates including humans, and the development of potential pathological treatments.

## Author Contributions

All authors listed have made a substantial, direct and intellectual contribution to the work, and approved it for publication.

### Conflict of Interest Statement

The authors declare that the research was conducted in the absence of any commercial or financial relationships that could be construed as a potential conflict of interest.
